# HOTTIP Enhances Gemcitabine and Cisplatin Resistance Through Sponging miR-637 in Cholangiocarcinoma

**DOI:** 10.3389/fonc.2021.664916

**Published:** 2021-07-05

**Authors:** Kun Gao, Shuhua Chen, Xiangyu Yang

**Affiliations:** ^1^ Department of Interventional Radiology, Beijing Chao-yang Hospital Affiliated with Capital Medical University, Beijing, China; ^2^ Department of Clinical Laboratory, Yunfu People’s Hospital, Yunfu, China

**Keywords:** cholangiocarcinoma, long non-coding RNAs, HOTTIP, miR-637, LASP1

## Abstract

Chemo-resistance prominently hampers the effects of systemic chemotherapy to cholangiocarcinoma (CCA). Long non-coding RNAs (lncRNAs) have been shown to have great importance not only in tumorigenesis but also in therapeutic prognosis. The aim of this study is to investigate the role of lncRNA HOTTIP in the chemo-resistance to cisplatin and gemcitabine (CG) in CCA. The upregulated expression of HOTTIP was observed in CCA patients and the upregulation was associated with therapeutic responsiveness and prognosis. HOTTIP silencing powerfully increased the chemotherapy sensitivity through weakening proliferation and colony formation and increasing apoptosis. Subsequently, miR-637 was identified as the functional target of HOTTIP, since mechanically it could be targeted by HOTTIP and functionally its overexpression dismissed the changes by HOTTIP silencing *in vitro* and *in vivo*. Moreover, LIM and SH3 domain protein 1 (LASP1) could be targeted and regulated by miR-637. In all, HOTTIP modulates the sensitivity to CG in CCA through the HOTTIP/miR-637/LASP1 regulatory axis, providing a new opportunities for CCA treatment.

## Introduction

Cholangiocarcinoma (CCA) is one kind of primary liver cancer originating from bile duct epithelial cells. It ranks after hepatocellular carcinoma in morbidity accounting for an approximate 15% of primary liver cancers. Unfortunately, CCA patients suffer very poor prognosis due to high malignancy (1). The majority of patients have lost their opportunity for radical surgery since they are diagnosed in the advanced stage. The standard systemic chemotherapy employing gemcitabine and cisplatin can only provide a modest therapeutic effect (2). It is urgently needed to expand the knowledge of the mechanisms modulating the chemotherapy responsiveness in cholangiocarcinoma (3).

Non-coding RNAs are a class of RNA transcripts with no protein-coding potential, whose important physiological and pathological role has been extensively characterized (4, 5). Among them, long non-coding RNAs (lncRNAs), which are defined as longer than 200 nucleotides, have been increasingly recognized to be important participators in regulating processes like cell cycle, transcriptional activation, and chromatin modification. Moreover, intensive evidence has shown that lncRNAs are deeply involved in tumorigenesis and progression of various cancers, and therefore, lncRNAs are regarded as potential targets for diagnosis and prognosis (6–12). HOXA transcript at the distal tip (HOTTIP) is a 3764 lncRNA, which is located at the 5′ tip of HOXA gene clusters. Studies have demonstrated that HOTTIP plays a vital role in activating several 5′ HOXA genes (13). HOTTIP functions as a signal transmitter from higher order chromosomal configuration into chromatin codes. Thus, it not only takes part in the regulation of development, but also displays an oncogenic role in multiple cancers like hepatocellular carcinoma, gastric cancer, and colorectal cancer. However, the role of HOTTIP is not fully investigated in cholangiocarcinoma.

Cisplatin and gemcitabine (CG) are drugs currently used as standard-of-care chemotherapy for patients with advanced CCA (2). Gemcitabine is a nucleoside analogue of cytidine and cisplatin contains antineoplastic drug C_l2_H_6_N_2_Pt that functions as an alkylating agent. The anti-tumor effects are achieved though inducing DNA strand breaks and preventing DNA repair. Interestingly, HOTTIP is a crucial oncogene, and one of its important functional methods is to regulate chromatin codes. The aim of this study is to investigate the role of HOTTIP in chemotherapy. We found HOTTIP was upregulated in CCA and the upregulation was negatively associated with chemotherapy therapeutic effects. HOTTIP silencing enhanced the sensitivity of CCA cells responding to CG *in vitro*. Functionally and mechanistically, HOTTIP was shown to induce chemo-resistance by sponging miR-637 and further regulating LASP1 expression. Briefly, our data demonstrate that HOTTIP plays a key role in chemotherapy resistance in CCA by sponging miR-637, which brings new methods to fight this disease.

## Methods

### Patient’s Tissues

CCA samples were collected from patients through biopsy or operation. No treatment including surgery or chemotherapy was administrated to the patients before sample collection. The study was initiated after approval by the ethics committee of Yunfu People’s Hospital. Serum was frozen at −80°C until use. The therapeutic effect evaluation referred to the mRECIST criteria where complete response (CR), partial response (PR), stable disease (SD), and progressive disease (PD) were defined.

### Cell Culture and Transfections

The human cholangiocarcinoma cell lines (QBC939 and CCLP-1) were obtained from the ATCC Cell Biology Collection (Rockville, USA). The cells passed the STR genotyping test before use. The medium consisted of Dulbecco’s Modified Eagle medium containing 10% fetal bovine serum (FBS), 100 U/ml of penicillin, and 1% streptomycin (Invitrogen). The HOTTIP siRNA (si-HOTTIP), negative control siRNA (si-NC), miR-637 inhibitor, negative control (NC) inhibitor, miR-637 mimics, negative control mimics, LASP1 mimics, and negative control mimics were purchased from Gene Pharma (Shanghai, China). Lipofectamine^®^2000 Reagent was employed to fulfil the transfections according to the instructions.

### RNA Extraction and Quantification

TRIzol reagent (Invitrogen) was employed to achieve the RNA extraction from tissues or cells. The concentration of RNA was quantified by NanoDrop ND-2000 (Thermo Fisher Scientific). After extraction, the RNA was applied to denaturing agarose gel electrophoresis to ensure integrity. The ABI StepOnePlus real-time PCR system (Applied Biosystem, Foster City, CA, USA) was utilized to conduct the quantitative real-time PCR. GAPDH was used as a reference and the 2^−ΔΔCT^ method was calculated for relative quantification. 

### Cell Proliferation Assay

The cell proliferative ability under the influence of CG was examined by a Cell Counting Kit-8 (CCK-8) assay. The CCA cells were seeded onto 96-well plates in a density of 10^4^ cells per well. The medium with CG (4 μM of cisplatin and 20 μM of gemcitabine) was administrated to the cells for 24 h. Then the CG medium was replaced with normal medium. Afterwards, the cells were incubated and tested by the CCK-8 kit at the time points of 0, 24, 48, 72, and 96 h. The fluorescent density at 450 nm was detected by an automatic microplate reader (Synergy4; BioTek, Winooski, VT).

### Colony Formation Assay

The colony formation ability of CCA cells under the influence of CG was tested. The silenced HOTTIP and negative control CCLP-1 and QBC939 cells were subjected to CG medium containing 4 μM of cisplatin and 20 μM of gemcitabine for 24 h. And then, the cells were trypsinized into separated ones and incubated with normal medium in 6-well plates for a week. The colonies were stained by 0.1% crystal violet and 20% methanol for analysis.

### Hoechst Staining

Apoptosis was detected by a Hoechst Staining Kit. The cells were incubated with methanol acetic acid for 10 min for fixation and then were stained with Hoechst 33258 (Beyotime, China) at 1 mg/ml. The staining was visualized under a fluorescence microscope (Nikon Corporation, Chiyoda-ku, Tokyo, Japan).

### Western Blot Assay

A Western blot assay was performed as previously described (14). The cells were lysed with a RIPA strong buffer (Beyotime, China) on ice. Then, a BCA protein assay kit (Thermo Fisher Scientific, USA) was used to measure the whole protein concentration. The protein was subjected to electrophoresis through a sodium dodecyl sulfate polyacrylamide gel. After being transferred onto the polyvinylidene fluoride membrane, it was blocked by 5% bovine serum albumin for 1 h. Then an overnight incubation by indicated primary antibodies was performed. Afterwards, the primary antibodies were detected with HRP-conjugated secondary antibodies (Santa Cruz, CA, USA) at 37°C for 1 h. Visualization of the proteins were accomplished by the enhanced chemiluminescence reagent (Santa Cruz, CA, USA). The primary antibodies of LASP1 and GAPDH were purchased from Abcam (Cambridge, UK).

### *In Situ* Hybridization Staining

The paraffin-embedded CCA samples were stained to measure the HOTTIP expression. The *in situ* hybridization (ISH) was performed following the instructions of the ISH kit (BOSTER, Wuhan, China). Briefly, the slices were removed of paraffin and then rehydrated. Then they were subjected to proteinase for 10 min at 37°C to get rid of the residual protein. The sections were washed with PBS three times and then were incubated with hybridization mix for 24 h at 40°C. Then blockage was achieved by a blocking buffer through a 30-min incubation. The slices were incubated with anti –DIG for 60 min. The DAB kit (Solarbio, Beijing, China) was employed to visualize the staining.

### Target Prediction

The targeting prediction of lncRNA HOTTIP with miR-637 and miR-637 with LASP1 was completed by TargetScan (http://www.targetscan.org).

### Dual Luciferase Reporter Assay

The luciferase reporter plasmids containing wild-type or mutant HOTTIP promoter were transfected into the QBC939 cells with Lipofectamine 2000 (Invitrogen) as narrated above. Forty-eight hours later, the firefly luciferase activity was detected by the Dual Luciferase Reporter Assay system (Promega). The intensities were normalized based on the Renilla luciferase activity.

### Pull-Down Assay

The QBC939 cells were transfected with Biotinylated miR-637 mimics or controls (50 nM). Forty-eight hours after transfection, the cells were collected and lysed with lysis buffer. After being lysed, streptavidin-coated magnetic beads (Pierce Biotechnology, Rockford, IL) were used to incubate the lysates to obtain the biotin-labeled RNA complex. The incubation was maintained at 4°C for 3 h. The bound RNA was purified with TRIzol LS (Life Technology). Then real-time PCR was performed to quantify the bound RNA.

### RNA Binding Protein Immunoprecipitation (RIP) Assay

The RIP assay was carried out according to the manufacturer’s instructions for the Magna RIP Kit (Millipore) and Ago2 antibody (Cell Signaling Technology). miR-637 mimics or control were transfected into the QBC939 cells as mentioned above. Forty-eight hours after transfection, the cells were washed with iced PBS and lysed with RIP lysis buffer. The lysates were subjected to 5 µg/L of primary antibodies at 4°C for 2 h. Then, prepared magnetic beads (50 µL) were applied to each sample and incubated for 24 h at 4°C. The beads were collected and washed with the RIP buffer five times. Subsequently, TRIzol LS (Life Technology) was used to resuspend the beads. Finally, the binding products were quantified by real-time PCR.

### *In Vivo* Mouse Model

All animal studies were conducted complying with the principles and procedures defined in the Guide for the Care and Use of Laboratory Animals (2011). The BALB/c mice (n=5, for each group) were subcutaneously injected with 5 × 10^6^ transfected QBC939 cells. One week post inoculation, cisplatin (2.5 mg/kg) and gemcitabine (25 mg/kg) were intraperitoneally administrated to the mice twice per week for three weeks. The growth of tumors was recorded by bioluminescence imaging. Once a week, the abdomens of mice were dehaired and the mice were intraperitoneal injected with D-luciferin (150 mg/kg). Then the mice were anesthetized and imaged 10 min after injection by using the Lumina II imaging system (Caliper Life Sciences, USA).

### Statistics

The quantitative data were expressed as mean ± standard error of the mean (SEM). The difference between two experimental groups was analyzed by the Student t test. The significant criteria is defined as p < 0.05.

## Results

### HOTTIP Was Upregulated in CCA

**Figure 1:**
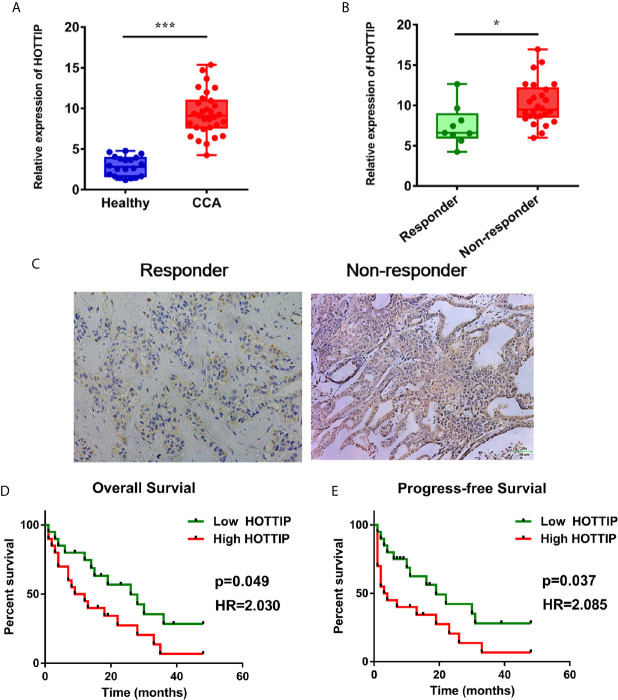
Upregulation of HOTTIP in CCA patients. **(A)** The serum HOTTIP expression levels of healthy volunteers and CCA patients. **(B)** The serum HOTTIP expression level CR+PR versus SD+PD patients before treatment. **(C)** *In situ* hybridization (ISH) staining of HOTTIP. Representative high and low HOTTIP expression images are shown. Overall survival **(D)** and disease-free survival **(E)** of high and low HOTTIP expression patients were analyzed by Kaplan-Meier analysis. (*p < 0.05, ***p < 0.001).

To begin with, the expression level of HOTTIP in CCA was explored. Similar to the results in other tumors, upregulation of HOTTIP in the serum of CCA patients was observed in comparison with healthy volunteers (**Figure 1A**). Then, we further investigated the HOTTIP expression profiles of patients with different chemotherapy responses. It turned out that the responders (CR and PR, n=9) held a significantly lower level of HOTTIP than the non-responders (SD and PD, n=31) (**Figures 1B, C**). Meanwhile, the patients were also divided into two groups according to the cut-off of medium HOTTIP level. The patients in the high HOTTIP group remained in a more advanced stage of disease (**Table 1**). The Kaplan-Meier survival curves illustrated that patients with higher HOTTIP expression possessed significantly worse overall survival and progression-free survival (**Figures 1D, E**).

**Table 1:** HOTTIP expression in CCA patients.

Variable	N	High expression	Low expression	P value
All cases	40	20	20	
Age (year)				0.741
>60	26	12	14	
<60	14	8	6	
Gender				0.342
Female	19	11	8	
Male	21	9	12	
Hepatitis				0.751
No hepatitis	21	11	10	
Hepatitis B	19	9	10	
Responsiveness				0.1274
PR+CR	9	2	7	
SD+PD	31	18	13	
Macroscopic growth patterns				0.749
Periductal infiltrating	23	12	11	
Mass forming	17	8	9	
Extent of disease				0.027
Locally advanced		7	14	
Lymph node metastasis and extrahepatic distant metastasis		13	6	
ECOG performance status				0.811
0		4	3	
1		7	6	
2		9	11	
CA19-9 Level				0.197
<200U/mL		6	10	
>200 U/mL		14	10	

### HOTTIP Silencing Enhanced the Sensitivity to Chemotherapy of CCA *In Vitro*

**Figure 2:**
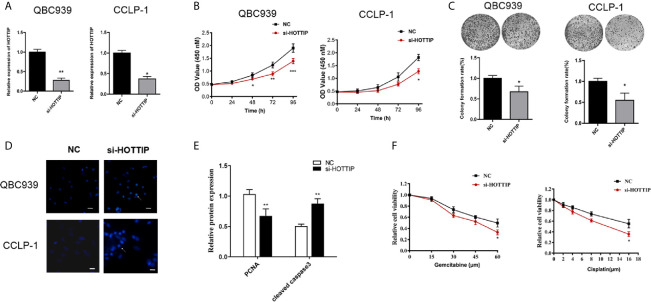
HOTTIP inhibition increased the susceptibility to chemotherapy of CCA *in vitro*. **(A)** The relative expression of HOTTIP quantified by qRT-PCR in CCA cell lines treated with the si-HOTTIP. **(B)** The proliferation of QBC939 and CCLP-1 cells. The si-NC or si-HOTTIP-transfected cells were pretreated with CG for 24 h before normal incubation. **(C)** The impact of HOTTIP inhibition on cell colony formation. Before the colony formation assay, CG pretreatment was performed. **(D)** Apoptosis was detected by Hoechst staining. **(E)** PCNA and cleaved caspase-3 expression was measured by qRT-PCR. **(F)** The viability of QBC939 and CCLP-1 cells was assessed by the CCK-8 assay. Tests were performed after treatments of different concentrations of cisplatin and gemcitabine. (*p < 0.05, **p < 0.01, ***p < 0.001).

To evaluate the impact of HOTTIP on chemo-resistance of CCA cells, a siRNA (si-HOTTIP) was developed to inhibit the HOTTIP expression (**Figure 2A**). The cell proliferation of CCA cells subjected to gemcitabine and cisplatin was assessed by a CCK-8 assay. It was found that under the influence of both gemcitabine and cisplatin, the growth rate of HOTTIP-silenced cells dramatically decreased in terms of the control cells (**Figure 2B**). The colony formation ability was also measured. Before the colony formation assay, the cells were pre-incubated with gemcitabine and cisplatin for 24 h. After a two-week incubation, the HOTTIP-silenced group developed fewer colonies than the control group (**Figure 2C**). Moreover, the influence of HOTTIP silencing on the apoptosis of the cells was also investigated. Hoechst staining detected more apoptotic nucleuses in the HOTTIP-silenced group (**Figure 2D**). Afterwards, proliferation and apoptosis was also profiled through quantification of protein markers. Proliferating Cell Nuclear Antigen (PCNA) is an important participator in DNA replication, so its expression is usually used to represent the activity of proliferation. Similarly, caspase-3 is an essential enzyme of apoptosis progress, and its active condition, cleaved caspase-3, is deemed as a marker of apoptosis. The PCNA and cleaved capspase-3 were quantified by real-time PCR. It was shown that the HOTTIP-silenced cells downregulated PCNA and upregulated cleaved capspase-3 (**Figure 2E**). The QBC939 and CCLP-1 cells were treated with CG in a dose-dependent manner. The results indicated that HOTTIP-silenced cells became more restricted with the enlargement of the CG concentration (**Figure 2F**). Taken together, when HOTTIP expression was inhibited, gemcitabine and cisplatin caused impaired proliferation and colony formation and magnified apoptosis in CCA cells, indicating that HOTTIP is an essential factor for CCA cells to resist chemotherapy.

### miR-637 Was a Direct Target of HOTTIP

**Figure 3:**
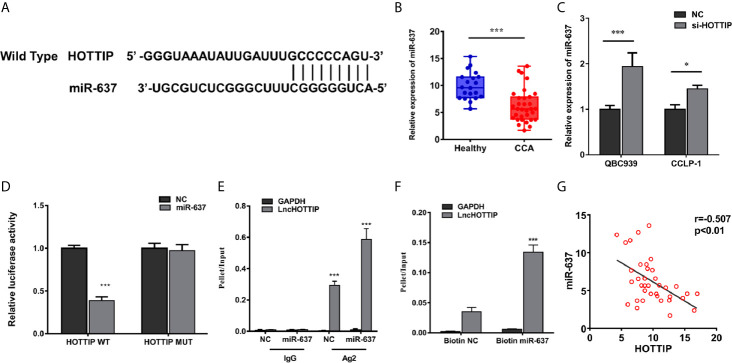
HOTTIP could bind to miR-637. **(A)** Predictive binding sites of lncRNA HOTTIP with miR-637. **(B)** The relative miR-637 expression levels of healthy volunteers and CCA patients. **(C)** The miR-637 expression level of HOTTIP inhibition in QBC939 and CCLP-1 cells. **(D)** Luciferase reporter gene assay of HOTTIP and miR-637. **(E)** The Anti-AGO2 immunoprecipitation (RIP) assay. The relative pellet/input ratios were calculated. **(F)** The pull-down assay of miR-637 and HOTTIP. **(G)** Correlation between HOTTIP and miR-637 in CCA patients. (*p < 0.05, ***p < 0.001).

As known, one of the most important mechanisms for lncRNAs of regulation in biological or pathological progresses is to sponge microRNAs (miRNAs). Target prediction was performed and miR-637 was indicated to be a potential target of HOTTIP (**Figure 3A**). Further, the relationship of HOTTIP and miR-637 was investigated. Firstly, the expression of miR-637 was quantified by qRT-PCR. miR-637 expression level in the serum of CCA patients was significantly lower than healthy volunteers (**Figure 3B**). Moreover, the impact of HOTTIP silencing on miR-637 was also detected in CCA cells. It was shown that CCA cells upregulated miR-637 expression when HOTTIP was silenced (**Figure 3C**). Furthermore, direct binding between HOTTIP and miR-637 was tested. The results of the luciferase reporter gene assay showed that overexpression of miR-637 decreased the luciferase activity of the cells with the HOTTIP reporter gene, while no significant change was found in the cells transfected with mut-HOTTIP (**Figure 3D**). The immunoprecipitation (RIP) assay demonstrated that a larger amount of HOTTIP was pulled down by miR-637 overexpressed cells compared with the normal controls (**Figure 3E**). What is more, in the pull-down assay, biotin-labeled miR-637 dragged more HOTTIP than negative controls (**Figure 3F**). In addition, a negative correlation between miR-637 and HOTTIP in CCA patients were found (**Figure 3G**). To sum up, it can be concluded that HOTTIP could directly interact with miR-637.

### miR-637 Inhibition Dismissed the Enhancement of Chemotherapy Sensitivity Induced by HOTTIP Silencing *In Vitro* and *In Vivo*

**Figure 4:**
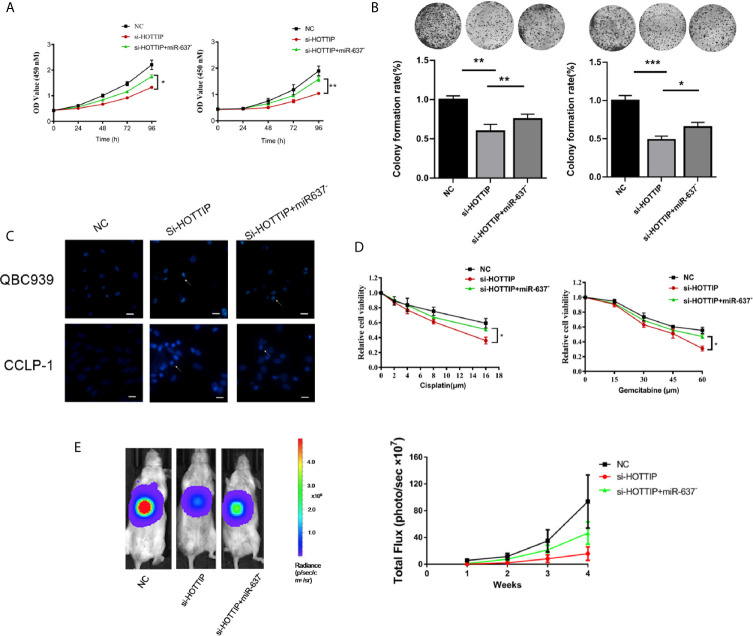
miR-637 inhibition dismissed the chemosensitivity enhancement induced by HOTTIP inhibition. The proliferation **(A)** and cell colony formation **(B)** of QBC939 and CCLP-1 cells. The si-NC, si-HOTTIP, and si-HOTTIP+ miR-637 inhibitor-transfected cells were pretreated with CG for 24 h before normal incubation. **(C)** Hoechst staining was used for apoptosis detection. **(D)** CG dose-dependent analysis of the three kinds of cells. **(E)** Bioluminescent imaging of the subcutaneous QBC939 tumor model. (*p < 0.05, **p < 0.01, ***p < 0.001).

Next, miR-637 was further investigated if it is a functional downstream target of HOTTIP. A series of rescue experiments were performed by inhibiting miR-637 in CCA cells. For proliferation evaluation, the cells with co-transfection of si-HOTTIP and miR-637 inhibitor grew much stronger than only si-HOTTIP-transfected cells (**Figure 4A**). Similarly, in a colony formation assay, the cells with HOTTIP and miR-637 co-inhibition formed more colonies than the controls with only inhibition of HOTTIP (**Figure 4B**). And then, apoptosis was also detected. CG-induced apoptosis was remarkably relieved by miR-637 inhibition (**Figure 4C**). We also measured the impact of miR-637 rescue in multiple concentrations of CG. The rescue effects became more significant to some extent (**Figure 4D**).

The functional roles of HOTTIP and miR-637 were further determined *in vivo*. A subcutaneous mouse model was established with local injection of luciferase-expressing CCA cells into the abdomen of mice. Once a week, gemcitabine and cisplatin were administrated by intraperitoneal injection and luciferase activity was recorded to monitor the growth status. Consistent with the *in vitro* studies, the HOTTIP-silenced tumor was mostly inhibited by the chemical drugs. Tumor growth inhibition was remarkably relieved with miR-637 inhibition (**Figure 4E**).

Overall, *in vitro* and *in vivo* experiments showed that the sensitivity to chemotherapy increased by HOTTIP silencing was essentially eliminated by miR-637 inhibition, suggesting that miR-637 should be an important functional target of HOTTIP.

### LASP1 Is Targeted by miR-637 to Regulate Chemo-Resistance

**Figure 5:**
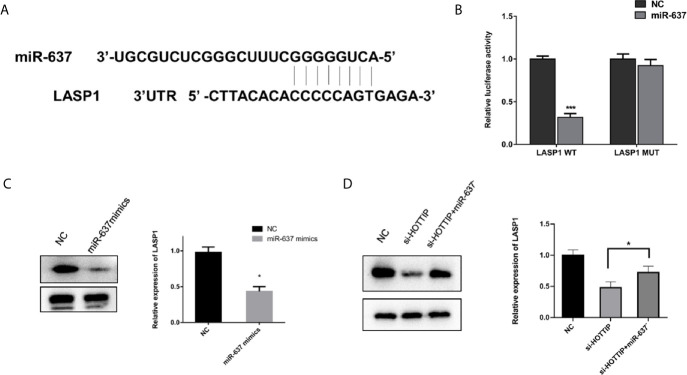
miR-637 regulated LASP1 expression. **(A)** Predictive binding sites of lncRNA HOTTIP with miR-637. **(B)** Luciferase reporter gene assay of miR637 and LASP1. **(C)** Western blot analysis of LASP1 expression under the influence of miR-637 overexpression in QBC939 cells. **(D)** Western blot analysis of LASP1 expression with HOTTIP inhibition and rescue of miR-637 inhibition. (*p < 0.05, ***p < 0.001).

LASP1 was brought into our sight by a prediction by starBase v2.0 (**Figure 5A**). Several studies have reported that LASP1 played a vital role in regulating the sensitivity to chemical drugs (15–17). The interaction between miR-637 and LASP1 was verified by the luciferase reporter gene assay. Concretely, LASP1 3′UTR reporter luciferase gene or its negative control sequence was transfected into the QBC939 cells. The cells with miR-637 overexpression strikingly decreased the bioluminescent activity in comparison with the normal control cells, while no evident changes were observed with the mutation of the binding motif bases of LASP1 3′UTR (**Figure 5B**). Then, the impact of miR-637 on the LASP1 expression was explored. With miR-637 upregulation, the LASP1 expression in QBC939 cells enormously decreased (**Figure 5C**). Further, a Western blot assay was performed to measure the LASP1 expression under HOTTIP silencing and rescue of miR-637 inhibition. As expected, the results showed that LASP1 expression level decreased with si-HOTTIP transfected into the CCLP-1 cells and the decreased LASP1 expression was reversed with miR-637 inhibition (**Figure 5D**). To sum up, it demonstrated that LASP1 is a mechanically and functionally downstream target of HOTTIP and miR-637, impacting the status of CCA cells.

## Discussion

Cholangiocarcinoma (CCA) is a kind of malignant tumor ranking second in primary liver cancers. Since the majority of CCA patients are unsuitable for surgery, chemotherapy remains the principal treatment for CCA. However, the treatment effect of chemotherapy is still very limited. Gemcitabine and cisplatin, the standard systemic chemotherapy regimen, are only slightly effective and provide a median overall survival of less than one year (2). It is, therefore, clinically meaningful to explore new targets for the deep understanding of the disease and to improve therapy response.

An increasing number of studies have focused on the regulation of chemo-resistance. For example, it was reported that microRNA-1249 could modulate the resistance to cisplatin and gemcitabine through changing CD133+ cell enrichment (13). Moreover, β-Caryophyllene was found to play an important role in the regulation of doxorubicin sensitivity and resistance *via* influencing STAT3 signaling, cell redox defenses, and cell cycling (18). In understanding the essential role of lncRNAs, the evidence of lncRNAs modulating chemosensitivity has emerged. For instance, lncRNA NEAT-1 was demonstrated to be an effector of regulating gemcitabine sensitivity. lncRNA HOTTIP is intensively reported to be an oncogene in a series of tumors regulating malignancy in multiple ways including impacting chemosensitivity (19–21). Therefore, attention was given to the significance of HOTTIP in CCA chemo-resistance. To answer this question, the HOTTIP expression profile in CCA and the impact caused by HOTTIP silencing were explored. The upregulation of HOTTIP and the chemosensitivity increase by HOTTIP silencing confirmed that HOTTIP was not only an oncogene for CCA development, but also a vital factor to resist chemotherapy.

Accumulating evidence has shown that lncRNAs could sponge miRNA to influence many biological and pathological processes (22). miR-637 has been intensively reported to play a crucial role in regulating growth, development, and oncogenesis in various kinds of cancers including cholangiocarcinoma (23–25). Specifically, miR-637 was indicated to be regulated by HOTTIP in papillary thyroid carcinoma (26). This research investigated the significance of miR-637 in influencing the chemotherapy response in CCA. To start with, the interaction between HOTTIP and miR-637 was enlightened by target prediction. Subsequently, the binding was verified by a luciferase reporter gene assay and RIP assay. Functionally, downregulation of miR-637 weakened the therapeutic effects of gemcitabine and cisplatin induced by HOTTIP inhibition *in vitro* and *in vivo*. So miR-637 was identified as a downstream target of HOTTIP to impact CCA chemo-responsiveness.

LASP1 was involved in the regulation of CCA chemo-responsiveness by HOTTIP/miR-637. It was reported that LASP1 is not only an important oncogene in AML but also in quite a few kinds of solid tumors (15, 17, 27). Interestingly, LASP1 was also reported to be regulated by microRNA. In the current study, it was found that LASP1 was not only targeted by miR-637 but was also regulated by HOTTIP and miR-637. So LASP1 is a functional target of HOTTIP/miR-637, which provides new points for settling CCA chemo-resistance.

In conclusion, the current study identified that upregulated HOTTIP expression in CCA patients was closely associated with chemo-responsiveness and subsequent clinical outcome. HOTTIP impacted the proliferation, colony formation, and apoptosis of the CCA cells under the influences of gemcitabine and cisplatin through the HOTTIP/miR-637/LASP1 regulatory axis. In short, the lncRNA HOTTIP and its regulatory pathway bring new opportunities for CCA treatment.

## Data Availability Statement

The data used to support the findings of this study are available from the corresponding author upon request.

## Ethics Statement

The studies involving human participants were reviewed and approved by the ethics committee of Yunfu People’s Hospital. The patients/participants provided their written informed consent to participate in this study. The animal study was reviewed and approved by Committee on the Use and Care on Animals of Capital Medical University.

## Author Contributions

XY and KG proposed the experimental design. KG, SC, and XY executed the experiments. XY composed the manuscript. The final manuscript was reviewed and approved by all authors before submission.

## Funding

This work was supported by the National Natural Science Foundation of China (81801753).

## Conflict of Interest

The authors declare that the research was conducted in the absence of any commercial or financial relationships that could be construed as a potential conflict of interest.

## Abbreviations

CCA, cholangiocarcinoma; lncRNAs, long non-coding RNAs; HOTTIP, HOXA transcript at the distal tip; LASP1, LIM and SH3 domain protein 1; ISH, *in situ* hybridization; RIP, RNA-binding protein immunoprecipitation; RECIST, Response Evaluation Criteria in Solid Tumors; PCNA, proliferating cell nuclear antigen.
